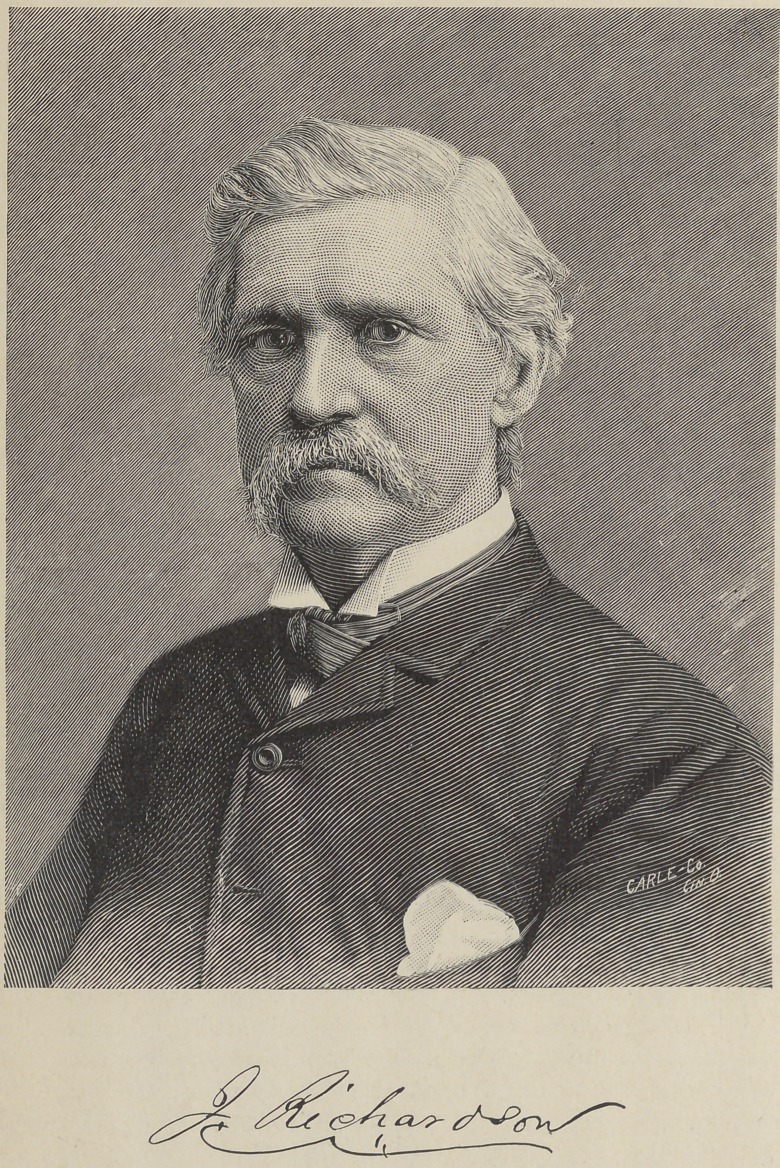# Biography of Dr. Joseph Richardson, D.D.S., M D.

**Published:** 1890-11

**Authors:** 


### THE DENTAL REGISTER.

VOL. XLIV.] NOVEMBER, 1890. [No. 11.

### BIOGRAPHICAL.

The recent death of Dr. Joseph Richardson, D.D.S., M.D., has made a profound impression throughout the profession of this country, and in that of other countries as well.

Dr. Richardson was born in Columbiana County, O., in 1824. His father's family settled in that part of the country about the year 1800. He there enjoyed an excellent opportunity for a general education, and especially so for that time. He recieved a classical education during his early life. He chose medicine as his profession. After a proper preliminary preparation for this, he took a course in the Ohio Medical College in 1847–48, after which he practiced for six years, and then began the study of dentistry. In 1853 he graduated at the Ohio College of Dental Surgery, in Cincinnati.

In March, 1853, Dr. Richardson was appointed Professor of Prosthetic Dentistry in his alma mater. This position he held about four years, and discharged the duties there devolving upon him with marked honor to himself, and to the great satisfaction of all interested. He was a most excellent teacher, systematic and thorough in the presentation of the subjects which he attempted to teach. He was always a great favorite with his classes. During this time, notwithstanding he was engaged in the practice of dentistry, he continued his study of medicine, and in 1854 he graduated in the Miami Medical College, and desiring to have the largest possible endorsement, he in 1859, entered the Ohio Medical College and took the M.D., from that institution. This he did, not from a mere desire to possess the largest possible endorsement, but from the determination to make himself most thorough in all the branches, either direct or collateral, that would aid him in his professional work.

During the time that he was professor in the Ohio College of Dental Surgery he wrote his practical treatise on "Mechanical Dentistry," which was published in 1860, a work that from that time to the present has remained as *the* text-book on this subject. It is the most thorough, extensive and best arranged work on this subject extant. It has passed through, in his owns hands, four editions, and has from the first to the present time been used as a text-book in all the dental colleges of this country and those of Europe as well. It has been translated into the German language. Up to 1869 it was the only text-book on the subject found in our dental colleges. So great was his interest in this work, and so intense was his application and labor in the preparation of the fourth edition that his health was greatly impaired thereby. From this enfeeblement he never recovered; that seemed to have been the beginning of his last illness. So that he literally sacrificed himself upon the altar of his chosen profession. He was a member of the American Dental Association, and in 1859 was one of a committee of three to draft a constitution for that society. He was a member of the Mississippi Valley Dental Association, and also of the Indiana State Dental Association, and of various similar organizations. When the Indiana Dental College was organized he was appointed to the chair of Mechanical Dentistry.

Up to the year 1862 he resided in Cincinnati, at least during his professional life. At that time he removed to Terre Haute, Ind., and there practiced his profession up to within three or four years.

As a man Dr. Richardson was well and favorably known, respected, and admired by all who knew him. In his community he occupied positions of trust and responsibility. He was specially interested in the education of the young; was connected with the city schools as a member of the Board of Directors for several years. And the duties pertaining to this office he faithfully discharged even in the time of his feeble health, and gave to it a trained intelligence, scholarly attainments, and an integrity that was unquestioned. All who came in contact with him became his friends, for he was a man of special attractive manners, and had in his conversation and in the trend of his mind a vein of kindly humor which gave a charm to all he said. As a father and husband he was a model man, loving his family with an intensity delightful to witness. In years past he had been greatly bereaved by the loss of three lovely children. This was a severe blow to him. He leaves a wife and two children, Laura and James. To them the sincere sympathy of his large professional acquaintance will be extended.

Dr. Richardson in his general course of life, in the love and devotion to the profession of his choice, stands as one for emulation, by all those of his profession who remain, and especially by the young whose characters are being formed and established. His memory will ever remain green with all who knew him, and his labors of love and devotion will be borne along upon the current of time to bless, not only this, but coming generations.

J. T.

---

To Bleach Rubber Dam.—Dr. George Brunton, England, says: Soak it in cold water and wrap it up. When dry it is white and will remain so for a considerable time.

To Stop Flow of Saliva, he uses a strong astringent known as chloralum which stopped the saliva for a couple of hours enabling him to work without the rubber dam.—*Dental Record.*

---

No Doctor Needed.—A wayfarer lately, in a primitive part of Kent, inquired of a rustic whom he met whether there was a doctor near as he had hurt his foot and wanted it looked to. "Doctor, sir?" said the man with a knowing shake of his head, "there ain't no such thing about here. If we sprains ourselves, or has the toothache, we goes to the blacksmith; but thank God we all dies natural deaths."—*Medical Record.*

---

Soft-Handed Sons of Toil is what Dr. Oliver Wendell Holmes calls the members of the medical profession.

## Figures and Tables

**Figure f1:**